# Effects of drug and hazardous alcohol use on having a detectable HIV viral load: An adherence mediation analysis

**DOI:** 10.1016/j.abrep.2023.100486

**Published:** 2023-03-11

**Authors:** Edward R. Cachay, Tesfaye S. Moges, Huifang Qin, Laura Bamford, David J. Grelotti, Wm. Christopher Mathews

**Affiliations:** a Department of Medicine, Division of Infectious Disease and Global Public Health, Owen Clinic, UC San Diego School of Medicine, United States; b HIV Neurobehavioral Research Program, Department of Psychiatry, UC San Diego School of Medicine, United States

**Keywords:** HIV viral load, Substance use, Adherence, Mediation analysis

## Abstract

**Objectives:**

People living with HIV (PWH) with substance or alcohol use often have unsuppressed plasma HIV viral loads (pVL). The degree to which substance and alcohol use effects on HIV viral suppression are mediated through medication nonadherence is incompletely understood.

**Methods:**

We included PWH prescribed antiretroviral therapy and receiving care at an academic HIV clinic between 2014 and 2018 who completed both patient-reported outcomes (PRO) questionnaires and had subsequent pVL measurements. Measures included assessments of alcohol use (AUDIT-C), drug use (NIDA-ASSIST), and self-reported adherence measured using four different methods. Substances found in bivariate analysis to predict detectable pVL were modeled separately for mediation effects through adherence. We report natural direct (NDE) and indirect effect (NIE), marginal total effect (MTE), and percentage mediated.

**Results:**

Among 3125 PWH who met eligibility criteria, 25.8% reported hazardous alcohol use, 27.1% cannabis, 13.1% amphetamines, 1.9% inhalants, 5.3% cocaine, 4.5% sedative-hypnotics, 2.9% opioids, and 2.3% hallucinogens. Excellent adherence was reported by 58% of PWH, and 10% had detectable pVL. Except for sedatives, using other substances was significantly associated with worse adherence. Bivariate predictors of detectable pVL were [OR (95% CI)]: amphetamine use 2.4 (1.8–3.2) and opioid use 2.3 (1.3–4.0). The percent of marginal total effect mediated by nonadherence varied by substance: 36% for amphetamine use, 27% for opioid use, and 39% for polysubstance use.

**Conclusion:**

Use of amphetamines, opioids, and multiple substances predicted detectable pVL. Up to 40% of their effects were mediated by self-reported nonadherence. Confirmation using longitudinal measurement models will strengthen causal inference from this cross-sectional analysis.

## Highlights

• People living with HIV (PWH) with substance use or alcohol use often have detectable plasma HIV viral loads that are predictive of negative clinical outcomes.
• Among PWH with substance or alcohol use, it is not known what proportion of the observed detectable HIV viremia is mediated through medication nonadherence.
• Use of amphetamines, opioids, and multiple substances but not alcohol predicted having a detectable HIV viral load.
• Up to 40% of the substance use effects were mediated by self-reported adherence.

## 1 Introduction

The potential negative effects of active alcohol and substance use on achieving and maintaining an undetectable HIV plasma HIV load (pVL) have been a concern since the advent of antiretroviral therapy. A common assumption is that any putative effects of alcohol or substance use on HIV viral control are mediated through effects on antiretroviral medication nonadherence (Amico et al., 2006, Chander et al., 2006, Hendershot et al., 2009). Regimens with higher resistance barriers, simplified dosing, and long half-lives may attenuate the detrimental effects of alcohol and substance use on adherence-mediated and adherence-independent causal pathways toward HIV viral load suppression (Christopoulos, Grochowski, Mayorga-Munoz, Hickey, Imbert, Szumowski, Dilworth, Oskarsson, Shiels, Havlir, & Gandhi, 2022).

Some studies have suggested the potential direct effects of how substance use (methamphetamine, cocaine, opioids, and cannabis) impacts immune activation pathways and facilitates HIV viral replication (Azzoni et al., 2020, Buch et al., 2020, Feldman et al., 2015, Jiang et al., 2016, Massanella et al., 2015, Napuri et al., 2013, Prasad et al., 2019, Rasbach et al., 2013, Saloner et al., 2020, Tyagi et al., 2016, Vidot et al., 2019). Alcohol use has also been associated with potentially detrimental effects on viral control, independent of medication adherence (Agudelo et al., 2015, Amedee et al., 2014, Bagasra et al., 1989, Molina et al., 2015).

Few studies have examined adherence-mediated and direct effects of hazardous alcohol and substance use on HIV viral suppression (Cook et al., 2017, Feelemyer et al., 2020, Kahler et al., 2017, Parsons et al., 2008). We hypothesized that the effects of alcohol and substance use on viral suppression would be largely mediated through effects on antiretroviral nonadherence. The primary study aim was to determine the proportion of the total effect of substance use (including alcohol) on having a detectable viral load that was mediated through antiretroviral adherence. Our secondary aim was to determine the proportion of patients having an undetectable pVL according to the frequency of substance use and self-reported adherence category.

## 2 Materials and methods

### 2.1 Study design and population

We conducted a retrospective cross-sectional analysis of persons with HIV (PWH) receiving care in an urban academic HIV clinic (Owen Clinic) at the University of California, San Diego (UCSD), in the United States, between 2014 and 2018. Our cohort included PWH 18 years of age or older with prescribed antiretroviral therapy who completed patient-reported outcome (PRO) questionnaires at least annually and had available the most recent, subsequent HIV pVL collected as part of routine care. Patients' characteristics included sex at birth, race, HIV risk factor, age, and study year and were collected using de-identified electronic medical records information. All participants signed a written consent form before enrolment in the study. The study protocol was approved by the UCSD Human Subjects Research Protection Program (UCSD HRRP #171338).

### 2.2 Data collected domains and procedures

The PRO questionnaire contains self-report items to ascertain alcohol use, substance use, and HIV antiretroviral adherence. Substance use measures included AUDIT-C and NIDA-ASSIST as measures of hazardous drinking over the past year (AUDIT-C ≥ 4 in men and ≥3 in women) and recent (within the past 3 months) substance use, respectively (Bradley et al., 2007, WHO Assist Working Group, 2002). Categories of prescription and recreational drugs surveyed in the PRO battery included cannabis, amphetamines (including methamphetamine), opioids (both legal and illegal), sedative-hypnotics, cocaine, inhalants, and hallucinogens. Self-report of adherence was assessed in four ways: (1) the number of doses missed in the last 7 days (1 = 0 missed, 6 = >4 missed); (2) Likert scaled adherence (1–6) where 1 = very poor and 6 = excellent; (3) visual analog scale (VAS) of percent adherence over the past month; and (4) last missed dose (1 = within the past week, 6 = never skip medications) (Amico et al., 2006, Feldman et al., 2013, Simoni et al., 2006, Stirratt et al., 2015).

### 2.3 Statistical methods

To select a single measure of medication adherence that most discriminated between those with detectable and undetectable follow-up HIV viral load measurements, we conducted receiver operating characteristic (ROC) analysis of the four candidate measures using binary coded viral load (≤200 copies/ml vs. >200 copies/ml) as the reference criterion. We chose 200 copies/ml as reference criteria as this value matches the threshold for lack of HIV transmission to uninfected partners (Eisinger, Dieffenbach, & Fauci, 2019). To evaluate whether the four adherence measures could jointly enhance discrimination beyond any individual measure, a logistic regression model was fit, and the predicted probabilities of viral non-suppression were estimated. ROC areas and Hosmer-Lemeshow tests were used to evaluate model discrimination and calibration, respectively. The optimal cut-point of the most discriminating predictor of viral non-suppression was estimated using Liu's method implemented in Stata *cutpoint.ado* (Liu, 2012), StataCorp. 2016. Stata Statistical Software: release 15.1. College Station, TX, USA.

The most discriminating adherence measure was then dichotomized at the optimal cut-point and used in subsequent analyses as the operationally defined measure of non-adherence. Next, we examined bivariate associations: (1) between alcohol and specific substance use and the most discriminating binary measure of adherence and (2) between alcohol and specific substance use and HIV viral non-suppression. We then examined the effects of alcohol and specific substance use on having a detectable pVL using unadjusted and mutually adjusted logistic regression models. Substances (including alcohol) that were independent predictors of having a detectable pVL in logistic models were subsequently selected as independent predictors of HIV viral non-suppression in the mediation analyses. Separate mediation models were fit for each selected substance using the Stata *paramed.ado* program to estimate the natural direct effect (NDE), natural indirect effect (NIE), and marginal total effect (MTE) on the odds ratio scale. Percent mediation was estimated as the ratio of the natural logarithms of odds ratios for the NIE/MTE (Emsley et al., 2013, Lee et al., 2021, Vanderweele and Vansteelandt, 2010). Sensitivity analysis controlling our mediation models for sex at birth, race, HIV transmission risk factor, and age was also conducted. We determined, based on the observed prevalence of unsuppressed pVL (10%), the prevalence of excellent adherence (58%), and the correlation between the drug or alcohol exposure measures and adherence varying from 0.1 to 0.5, that with type I error = 0.05 and power = 0.8, we could detect a mediation odds ratio from 1.4 to 1.49, respectively, with a sample size of 3000 (Vittinghoff, Sen, & McCulloch, 2009).

## 3 Results

Table 1 shows the characteristics of the study participants. During the study period, 3125 patients met the eligibility criteria. PWH had a median age of 51 years, of whom 54% were non-white, 12% were female, and 72% were men who have sex with men (MSM) as self-reported HIV risk factors. The prevalence of hazardous alcohol consumption was 26%, and use of prescription and recreational substances was common: marijuana (27%), amphetamine (13%), inhalants (12%), sedative-hypnotics (5%), and opioids (3%). Multiple substance use was reported by 17% of participating PWH. The mean time from PRO to HIV viral load collection was 89 days (SD: 155 days). Despite prevalent substance use, most (90%) patients had undetectable HIV pVL (<200 copies/ml), and more than half (58%) reported excellent adherence to antiretroviral therapy.

**Table 1:** Patient characteristics.

Sex	
Male	2742 (87.7%)
Female	383 (12.3%)

Race	
White	1450 (46.4%)
Latino	1025 (32.8%)
Black	417 (13.3%)
Asian	121 (3.9%)
Other	112 (3.6%)

HIV Risk Factor	
MSM, not IDU	2036 (65.2%)
IDU, not MSM	132 (4.2%)
IDU + MSM	208 (6.7%)
Heterosexual, not-IDU	536 (17.2%)
All others	213 (6.8%)
Age, median (IQR)	51.0 (41.0–58.0)
Age, mean (SD)	50.1 (11.7)

Year	
2014	378 (12.1%)
2015	427 (13.7%)
2016	574 (18.4%)
2017	738 (23.6%)
2018	1008 (32.3%)

Amphetamines	
No	2620 (86.9%)
Yes	395 (13.1%)
MISSING	110 observations

Sedatives	
No	2813 (95.5%)
Yes	133 (4.5%)
MISSING	179 observations

Cocaine	
No	2810 (94.7%)
Yes	158 (5.3%)
MISSING	157 observations

Opioids	
No	2884 (97.1%)
Yes	86 (2.9%)
MISSING	155 observations

Marijuana	
No	2177 (72.9%)
Yes	810 (27.1%)
MISSING	138 observations

Hallucinogens	
No	2867 (97.7%)
Yes	66 (2.3%)
MISSING	192 observations

Inhalants	
No	2594 (88.1%)
Yes	349 (11.9%)
MISSING	182 observations

AUDIT-C Score, median (IQR)	1.0 (0.0–3.0)
MISSING	107 observations

AUDIT-C: Hazardous Drinking	
Not Hazardous Drinking	2240 (74.2%)
Hazardous Drinking	778 (25.8%)
MISSING	107 observations

Self Rated Adherence	
<Excellent	1314 (42.3%)
Excellent	1796 (57.7%)
MISSING	15 observations

Self Rated Adherence (VAS)	
<=98	927 (55.7%)
>98	736 (44.3%)
MISSING	1462 observations

Interval of last missed dose	
2–4 weeks, 1–2 weeks, or past week	678 (39.5%)
1–3 months, >3 months, or never	1040 (60.5%)
MISSING	1407 observations

Number of Missed Doses (7 days)	
≤1 missed dose	999 (69.7%)
>1 missed dose	434 (30.3%)
MISSING	1692 observations

Detectable Plasma Viral Load (pVL)	
Undetectable (<200c/ml)	2820 (90.2%)
Detectable (≥200c/ml)	305 (9.8%)
pVL, median (IQR)	40 (20,40)
Log_10_ (pVL), median (IQR)	1.6 (1.30, 1.60)
Days between PRO date & pVL date, median (IQR)	24.0 (2.0–120.0)
MISSING	12 observations
Days between PRO date & pVL date, mean (SD)	88.8 (154.8)
MISSING	12 observations

No Substances Used (ASSIST + AUDIT)	
0	1610 (59.8%)
1	633 (23.5%)
2	281 (10.4%)
3	103 (3.8%)
4–7	67 (2.5%)
MISSING	431 observations
No Substances Used (ASSIST + AUDIT), median (IQR)	0.0 (0.0–1.0)
MISSING	431 observations
No Substances Used (ASSIST + AUDIT), mean (SD)	0.7 (1.0)
MISSING	431 observations

MSM: men having sex with men; IDU: injectable drug use; VAS: visual analogue scale; IQR: interquartile range; SD: standard deviation; PRO: patient-reported outcomes.

Table 2 presents the measurement properties of the four single-item adherence measures and the composite predicted adherence proportion based on logistic regression of the four single items on HIV pVL. Because the fewest non-missing values (n = 3110) were with item 2 and because the pVL discrimination (ROC area) of this item was highest among the single-item adherence measures, we chose this item for use in mediation analysis. The optimal antiretroviral adherence cut-point in predicting having a detectable pVL was at excellent (6) vs. not excellent (≤5). An adherence score ≤5 on this item was operationally defined as non-adherence.

**Table 2:** Patient-reported outcomes (PRO) measures of antiretroviral medication adherence: measurement properties.

Item	Content	Possible Score Range	Observed Score Range	Observed Score Median [Mean]	N (observed)	N(missing)	ROC Area _2_	Optimal Cut point _2_
1.	# of doses missed (7 days)	1 = 0 missed; 6= >4 missed	1- 6	1 [1.6]	1433	1692	0.65	>1
2.	Self Rated Adherence	1 = very poor; 6 = excellent	1- 6	6 [5.2]	3110	15	0.71	>5
3.	Self Rated Adherence Visual Analogue Scale (VAS)	0--100	0--100	98 [92.3]	1663	1,462	0.70	>98
4.	Last Missed Dose	1 = past week; 6 = never miss	1- 6	4 [3.9]	1718	1407	0.62	>3
5. Composite_1_	Composite logistic regression predictor of viral non-suppression	0–1.0	0.05--0.77	0.06 [0.10]	1374	1751	0.73	>0.078

1. Composite predicted adherence proportion based on logistic regression of items 1–4 on viral load indicator.

2. For items 2–4, undetectable viral load (<200 copies/ml) was coded 1. For items 1 and 5, undetectable viral load was coded 0.

Table 3 presents the substance and pVL distributions according to the self-reported adherence category. Nonadherence was significantly associated with detectable pVL and the use of amphetamines, cocaine, opioids, marijuana, hallucinogens, and inhalants. Table 4 shows substance use and self-reported adherence according to pVL category. Having a detectable pVL was significantly associated with nonadherence and the use of amphetamines and opioids. In contrast, hazardous alcohol consumption and the use of marijuana, cocaine, inhalants, sedative-hypnotics, and hallucinogens were not predictive of detectable pVL.

**Table 3:** Distribution of substance use by adherence level.

Factor	Level	No Excellent adherence	Excellent adherence	p-value	Test
N		1323	1802		
Amphetamines	No	1006 (79.5%)	1614 (92.3%)	<0.001	Fisher's exact
	Yes	260 (20.5%)	135 (7.7%)		
Sedatives	No	1163 (95.0%)	1650 (95.8%)	0.32	Fisher's exact
	Yes	61 (5.0%)	72 (4.2%)		
Cocaine	No	1150 (93.0%)	1660 (95.9%)	<0.001	Fisher's exact
	Yes	87 (7.0%)	71 (4.1%)		
Opioids	No	1186 (95.9%)	1698 (98.0%)	0.001	Fisher's exact
	Yes	51 (4.1%)	35 (2.0%)		
Marijuana	No	848 (68.1%)	1329 (76.3%)	<0.001	Fisher's exact
	Yes	398 (31.9%)	412 (23.7%)		
Hallucinogens	No	1180 (96.8%)	1687 (98.4%)	0.005	Fisher's exact
	Yes	39 (3.2%)	27 (1.6%)		
Inhalants	No	1054 (86.5%)	1540 (89.3%)	0.021	Fisher's exact
	Yes	165 (13.5%)	184 (10.7%)		
AUDIT-C Score, median (IQR)		1 (0, 4)	1 (0, 3)	0.001	Wilcoxon rank-sum
AUDIT-C: Hazardous Drinking	Not Hazardous Drinking	902 (71.2%)	1338 (76.4%)	0.002	Fisher's exact
	Hazardous Drinking	364 (28.8%)	414 (23.6%)		
Detectable pVL (≥200c/ml)	Undetectable (<200c/ml)	1101 (83.2%)	1719 (95.4%)	<0.001	Fisher's exact
	Detectable (>=200c/ml)	222 (16.8%)	83 (4.6%)		
No Substances Used (ASSIST + AUDIT-C)	0	590 (53.2%)	1020 (64.3%)	<0.001	Fisher's exact
	1	281 (25.4%)	352 (22.2%)		
	2	136 (12.3%)	145 (9.1%)		
	3	64 (5.8%)	39 (2.5%)		
	4–7	37 (3.3%)	30 (1.9%)		
No Substances Used (ASSIST + AUDIT), mean (SD)		0.81 (1.07)	0.55 (0.90)	<0.001	Two sample *t* test
No Substances Used (ASSIST + AUDIT), median (IQR)		0 (0, 1)	0 (0, 1)	<0.001	Wilcoxon rank-sum

pVL: HIV plasma viral load; IQR: interquartile range; SD: standard deviation.

**Table 4:** Distribution of substance use by HIV viral suppression category.

Factor	Level	Undetectable (<200c/ml)	Detectable (≥200c/ml)	p-value	Test
N		2820	305		
Amphetamines	No	2403 (88.1%)	217 (75.6%)	<0.001	Fisher's exact
	Yes	325 (11.9%)	70 (24.4%)		
Sedatives	No	2552 (95.5%)	261 (95.3%)	0.88	Fisher's exact
	Yes	120 (4.5%)	13 (4.7%)		
Cocaine	No	2546 (94.7%)	264 (94.3%)	0.78	Fisher's exact
	Yes	142 (5.3%)	16 (5.7%)		
Opioids	No	2622 (97.4%)	262 (94.2%)	0.007	Fisher's exact
	Yes	70 (2.6%)	16 (5.8%)		
Marijuana	No	1983 (73.3%)	194 (69.0%)	0.14	Fisher's exact
	Yes	723 (26.7%)	87 (31.0%)		
Hallucinogens	No	2607 (97.9%)	260 (96.3%)	0.13	Fisher's exact
	Yes	56 (2.1%)	10 (3.7%)		
Inhalants	No	2360 (88.3%)	234 (87.0%)	0.55	Fisher's exact
	Yes	314 (11.7%)	35 (13.0%)		
AUDIT-C Score, median (IQR)		1 (0, 3)	1 (0, 3)	0.64	Wilcoxon rank-sum
AUDIT-C: Hazardous Drinking	Not Hazardous Drinking	2020 (74.2%)	220 (74.3%)	1.00	Fisher's exact
	Hazardous Drinking	702 (25.8%)	76 (25.7%)		
ART Adherence	No Excellent adherence	1101 (39.0%)	222 (72.8%)	<0.001	Fisher's exact
	Excellent adherence	1719 (61.0%)	83 (27.2%)		
No Substances Used (ASSIST + AUDIT), mean (SD)		0.64 (0.97)	0.86 (1.09)	<0.001	Two sample *t* test
No Substances Used (ASSIST + AUDIT), median (IQR)		0 (0, 1)	0 (0, 1)	<0.001	Wilcoxon rank-sum

IQR: interquartile range; SD: standard deviation.

In both unadjusted and mutually adjusted logistic regression models, the independent use of amphetamines and opioids was significantly associated with having a detectable pVL (Table 5). PWH reporting amphetamine or opioid use were more than twice as likely to have a detectable pVL. The inability of binary-coded hazardous alcohol use to predict having a detectable pVL was confirmed in ROC analysis of the numeric AUDIT-C score evaluated against pVL as reference criterion (ROC 0.492, 95% C. I. 0.457–0.527).

**Table 5:** Unadjusted and adjusted effects of drug and alcohol use on detectable HIV viral load.

Predictor	Unadjusted Effects			Mutual Adjusted Effects_1_		
	Odds Ratio	95% CI_2_	p-value	Odds Ratio	95% CI	p-value
Sedatives	1.059	0.589, 1.904	0.847	0.693	0.352, 1.366	0.29
Cocaine	1.087	0.638, 1.850	0.76	0.568	0.287, 1.124	0.104
Amphetamines	2.385	1.779, 3.198	0.0001	2.166	1.509, 3.107	<0.0001
Opioids	2.287	1.310, 3.996	0.004	2.173	1.157, 4.081	0.016
Marijuana	1.23	0.942, 1.606	0.128	1.028	0.757, 1.396	0.862
Hallucinogens	1.791	0.903, 3.551	0.095	1.46	0.65, 3.281	0.359
Inhalant	1.124	0.773, 1.634	0.54	0.807	0.521, 1.25	0.337
Alcohol: Hazardous	0.994	0.755, 1.308	0.966	1.044	0.767, 1.421	0.783

1. Hosmer-Lemeshow chi-square (7 d.f.) = 4.03, p = 0.545. ROC area = 0.591.

2. CI: confidence interval.

Table 6 presents mediation model results for substances that were found to be significant predictors of having a detectable pVL. For single substances, the percent mediation was highest (36%) for amphetamine use, while for opioids, the percent mediated was 27%. The percentage mediation for multiple substance use was 40%. After controlling for sex at birth, race, HIV transmission risk factor, and age, the percent mediation for amphetamine, opioids, and multiple substances did not change substantially and were 38%, 28%, and 40%, respectively (Supplementary Table). Examining missing value patterns across all variables included in any mediation analyses presented in Table 6, those with no missing values (complete cases, n = 2936) did not differ from those with one or more missing values (incomplete cases, n = 189) by year of measurement, sex, or age (p > 0.05). However, complete cases were more likely to identify as white versus non-white (47% vs. 37%, p = 0.002) and to report male sex with men and not injection drug use (MSM, not-IDU) as their HIV transmission risk factor (66% vs. 56%, p = 0.01).

**Table 6:** Mediation analysis results.

		Effect Odds Ratio	Bootstrap Std Error	95% C.I.	
Amphetamines					
	NDE	1.75	0.28	1.26	2.38
	NIE	1.37	0.05	1.29	1.48
	MTE	2.40	0.38	1.75	3.31
	Percent Mediated	36.18%			

Opioids					
	NDE	1.86	0.60	0.86	2.99
	NIE	1.25	0.08	1.11	1.41
	MTE	2.33	0.75	1.10	3.83
	Percent Mediated	26.48%			

No. Substances Reported					
	NDE	1.13	0.07	0.98	1.28
	NIE	1.09	0.02	1.06	1.12
	MTE	1.23	0.08	1.07	1.39
	Percent Mediated	39.74%			

NDE: natural direct effect, NIE: natural indirect effect, MTE: marginal total effect.

## 4 Discussion

Our research found that recent use of amphetamines, cocaine, opioids, marijuana, hallucinogens, inhalants, hazardous alcohol use, and multiple substance use were significantly associated with less than excellent self-reported antiretroviral adherence. Although with variability in their conclusions, many studies have previously documented the detrimental effects of the use of specific recreational substances and alcohol on various adherence measures, including self-report and electronic monitoring approaches (Chander et al., 2006, Hendershot et al., 2009, Gonzalez et al., 2011, Lai et al., 2020, Rosen et al., 2013, Socias and Milloy, 2018, Velloza et al., 2020). Most robust conclusions pertain to the use of amphetamines, cocaine, opioids, and alcohol. Whereas for cannabis use, the intensity and regularity of use may modify the effects on adherence (Bonn-Miller et al., 2014, Vidot et al., 2017).

### 4.1 Strengths and limitations

Our study focus was on distal and more impactful outcomes of antiretroviral therapy, specifically HIV viral suppression. Self-reported use of amphetamines, opioids as individual substances and multiple substance use predicted having a detectable pVL. However, surprisingly hazardous alcohol use was not predictive.

Our study found that hazardous alcohol use was associated with worse ART adherence but not with having detectable HIV pVL as others have noted (Nolan et al., 2017). A large *meta*-analysis also found a significant and reliable association between alcohol use and antiretroviral medication nonadherence; however, it did not address the effects of alcohol on viral suppression (Hendershot et al., 2009). Important differences in study design, secular trends in the type of antiretroviral therapy, and instruments used to measure alcohol use could account for differences in our findings from other studies that observed hazardous alcohol use to be associated with detectable viral loads. Some were based on cross-sectional assessments (Conen et al., 2009). The ones with the longitudinal design were conducted in the late 1990s to early 2000s when antiretroviral therapy was poorly tolerated, requiring a significant pill burden, and many PWH with hazardous alcohol use avoided taking them while drinking (Chander et al., 2006, Conigliaro et al., 2003). Our study, conducted in a more contemporary antiretroviral era, suggests that, despite imperfect adherence, PWH with hazardous alcohol use can take enough medications to keep their pVL undetectable. Nonetheless, we did not assess patterns of alcohol intake which have been shown to impact pVL, with those with binge drinking having more likelihood of having detectable pVL (Conigliaro et al., 2003). Like our findings, a prospective randomized controlled trial failed to show the same association between hazardous alcohol use and detectable pVL (Samet et al., 2005). Noteworthy, in many of these cohorts, either exposure was defined as any alcohol use or instruments other than AUDIT-C were used (Palepu et al., 2003). We believe that our inability to detect an effect of hazardous alcohol use on pVL was not attributable to the specific AUDIT-C cutoff score used. We assessed, using ROC analysis, the full numeric range of AUDIT-C scores and found that the measure did not discriminate between those with and without HIV viral suppression.

This study found that up to 40% of the effects of having a detectable pVL appeared to be mediated by our measure of medication adherence. Thus, mediation analyses restricted to substances that were associated with having a detectable pVL (amphetamines, opioids, and multiple substance use) did not support our *a priori* hypothesis that their effects on viral suppression would be largely mediated through antiretroviral adherence. There are several potential explanations for this observation. This could represent a true finding, pointing to the effect of these substances not mediated through antiretroviral adherence. Another potential explanation is the occurrence of pharmacokinetic or pharmacodynamic drug-drug interactions that adversely influence antiretroviral effects independent of adherence behavior (Desai et al., 2020). Other possible explanations are related to measurement issues: the specific metric chosen for substance use (any past 3-month use on NIDA ASSIST), the timing of the substance use recall period with respect to subsequent viral load measurement (median 24 days), and use of an insensitive measure of medication adherence (although we selected the most discriminating of the candidate measures available (Table 2). Residual confounding and collider effects resulting in selection bias owing to missing values must also be considered. These issues merit further exploration and replication.

Among the few published reports of mediation analyses examining the effects of alcohol or substance use on viral non-suppression, we identified 3 concerning effects of alcohol or methamphetamine use. All three reported that the predominant effects were direct and not mediated through selected measures of adherence. Kahler et al. used a longitudinal cohort design (n = 533 patients) to examine the extent to which the effect of 30-day heavy drinking on viral non-suppression was mediated by antiretroviral adherence (measured by the number of missed doses over a 3-day period) (Kahler et al., 2017). These investigators found a significant natural indirect effect (NIE) on the risk ratio scale (1.03 [95% CI: 1.00–1.05]), corresponding to a 20 percent mediation effect. Cook et al. reported results of a mediation analysis using baseline data (n = 619 patients) for which the adherence measure was based on a 30-day number of days with missing doses and drinking measures included specifications for low, binge, and heavy drinking according to AUDIT-C criteria (Cook et al., 2017). For heavy drinking, the predominant effect on viral non-suppression was direct, with only 9.1% mediated through adherence. We were able to identify only one mediation analysis for the effects of methamphetamine use and antiretroviral adherence on HIV viral non-suppression (Feelemyer et al., 2020). Employing a cohort design (n = 645 patients), the investigators reported that the effect of methamphetamine use was predominantly direct. Unfortunately, the indirect effect was not reported. So, our findings of the predominant direct effects of amphetamine, opioid, and polysubstance use on viral non-suppression are consistent with previous reports.

Limitations of our findings are that they represent a single-center experience, a cross-sectional rather than a longitudinal design, and are based on a data set with missing values that limited complete case analysis for multivariable components of the analysis. The self-report nature of substance use and adherence might have potentially impacted the observed associations. Often, self-report substance use underestimates the use (Khalili et al., 2021), and self-reported adherence tends to overestimate adherence (Stirratt et al., 2015). Also, participant characteristics linked to social determinants of health, such as being unsheltered, mental health, and poverty, were not included in the analysis; hence, their contribution to observed effects could not be explored in this study. Our analysis mainly included white individuals and MSM, and our results might not be generalizable to HIV cohorts with more diverse characteristics. Nonetheless, we conclude that the mediation analysis findings are provocative and merit examination in expanded longitudinal cohorts with longitudinal mediation modeling (O'Laughlin, Martin, & Ferrer, 2018).

### 4.2 Conclusion

In summary, in a cohort of 3125 PWH, the use of amphetamines, opioids, and multiple substances predicted a detectable plasma HIV viral load. Up to 40% of the effects were mediated by self-reported adherence.

## Funding

This study is unfunded. The Clinical Investigation Core of the San Diego Center for AIDS Research (CFAR) provided administrative assistance in retrieving data (AI036214).

## Data Access Statement

Data supporting this study are not publicly available but can be accessed upon request. Please contact the corresponding author: ecachay@health.ucsd.edu.

## Role of the sponsor

The sponsor had no role in the design and conduct of the study; collection, management, analysis, and interpretation of the data; preparation, review, or approval of the manuscript; and decision to submit the manuscript for publication.

## Previous presentation

None.

## CRediT authorship contribution statement

**Edward R. Cachay:** Conceptualization, Methodology. **Tesfaye S. Moges:** Conceptualization, Writing – original draft, Writing – review & editing, Methodology. **Huifang Qin:** Data curation. **Wm. Christopher Mathews:** Supervision, Conceptualization, Writing – original draft, Formal analysis, Software, Writing – review & editing.

## Declaration of Competing Interest

The authors declare the following financial interests/personal relationships which may be considered as potential competing interests: **EC** received research grant funding paid to the Regents of the University of California from Gilead and Merck, and consulting fees for an advisory board from Gilead for unrelated projects. The other authors have no reported conflicts of interest.
