# Supplementary material for: Effects of drug and hazardous alcohol use on having a detectable HIV viral load: An adherence mediation analysis
Source: Addict Behav Rep. 2023 Mar 11;17:100486. doi: 10.1016/j.abrep.2023.100486 (PMC10025983; doi:10.1016/j.abrep.2023.100486)
Supplement: Supplementary data 1 [file mmc1.docx]

| **Supplementary Table: Mediation analyses adjusted for demographic characteristics** | | | | | |
| --- | --- | --- | --- | --- | --- |
|  |  | **Effect Odds Ratio** | **Bootstrap Std Error** | **95% C.I.** | |
| **Amphetamines** |  |  |  |  |  |
|  | **NDE** | 1.75 | 0.29 | 1.25 | 2.47 |
|  | **NIE** | 1.38 | 0.05 | 1.29 | 1.49 |
|  | **MTE** | 2.43 | 0.40 | 1.72 | 3.42 |
|  | **Percent Mediated** | 37.52% |  |  |  |
| **Opioids** |  |  |  |  |  |
|  | **NDE** | 1.76 | 0.59 | 0.77 | 2.92 |
|  | **NIE** | 1.25 | 0.08 | 1.10 | 1.41 |
|  | **MTE** | 2.19 | 0.74 | 0.96 | 3.82 |
|  | **Percent Mediated** | 28.22% |  |  |  |
| **No. Substances Reported** |  |  |  |  |  |
|  | **NDE** | 1.14 | 0.08 | 0.98 | 1.28 |
|  | **NIE** | 1.10 | 0.02 | 1.07 | 1.14 |
|  | **MTE** | 1.25 | 0.09 | 1.08 | 1.42 |
|  | **Percent Mediated** | 39.74% |  |  |  |

NDE: natural direct effect, NIE: natural indirect effect, MTE: the marginal total effect

Mediation models adjusted for sex, race, HIV transmission risk factor, and age
